# *Serratia marcescens* Is Able to Survive and Proliferate in Autophagic-Like Vacuoles inside Non-Phagocytic Cells

**DOI:** 10.1371/journal.pone.0024054

**Published:** 2011-08-25

**Authors:** Griselda V. Fedrigo, Emanuel M. Campoy, Gisela Di Venanzio, María Isabel Colombo, Eleonora García Véscovi

**Affiliations:** 1 Instituto de Biología Molecular y Celular de Rosario (IBR-CONICET), Departamento de Microbiología, Facultad de Ciencias Bioquímicas y Farmacéuticas, Universidad Nacional de Rosario, Rosario, Argentina; 2 Laboratorio de Biología Celular y Molecular-Instituto de Histología y Embriología (IHEM), Facultad de Ciencias Médicas, Universidad de Cuyo, CONICET, Mendoza, Argentina; University of Birmingham, United Kingdom

## Abstract

*Serratia marcescens* is an opportunistic human pathogen that represents a growing problem for public health, particularly in hospitalized or immunocompromised patients. However, little is known about factors and mechanisms that contribute to *S. marcescens* pathogenesis within its host. In this work, we explore the invasion process of this opportunistic pathogen to epithelial cells. We demonstrate that once internalized, *Serratia* is able not only to persist but also to multiply inside a large membrane-bound compartment. This structure displays autophagic-like features, acquiring LC3 and Rab7, markers described to be recruited throughout the progression of antibacterial autophagy. The majority of the autophagic-like vacuoles in which *Serratia* resides and proliferates are non-acidic and have no degradative properties, indicating that the bacteria are capable to either delay or prevent fusion with lysosomal compartments, altering the expected progression of autophagosome maturation. In addition, our results demonstrate that *Serratia* triggers a non-canonical autophagic process before internalization. These findings reveal that *S. marcescens* is able to manipulate the autophagic traffic, generating a suitable niche for survival and proliferation inside the host cell.

## Introduction

*Serratia marcescens* is a wide host range enterobacterium that can be isolated from plants, insects and nematodes, also being an opportunistic pathogen of mammals. In humans, *S. marcescens* is commonly isolated from urinary tract infections, nosocomial pneumonia, surgical wounds, and bloodstream infections, mostly in intensive care unit patients. The incidence of *S. marcescens* infections has increased over the last years. This emergence is mainly attributed to the acquisition of multiple antibiotic resistances and to the ability of the bacteria to adhere and persist on unanimated surfaces [1]–[3]. However, few reports have explored in depth the mechanisms that *Serratia* uses to invade, survive and proliferate in the host.

Hertle and Schwartz [4] have previously shown that *S. marcescens* is able to invade epithelial cell lines. Supporting evidence that *Serratia* is able to invade non-phagocytic cells has been provided by Neheme and co-workers who found that *Serratia* invaded the *Drosophila melanogaster* mid-gut epithelial cells when it transverses the gut to reach the body cavity [5]. Besides, a multi-host screening performed in *Caenorhabditis elegans*, *D. melanogaster* and culture epithelial cells demonstrated the capacity of the bacteria to display pathogenic traits in a broad range of hosts [6].

Following their uptake into a host cell membrane-bound compartment, bacterial pathogens face the challenge posed by the intracellular innate defense responses. One of the most conspicuous removal mechanisms depends on the endocytic pathway. Shortly after entry, the bacterium is wrapped in a host-cell derived phagocytic compartment. This vesicle is expected to suffer a maturation process from early to late phagosome and fuse with lysosomes, turning into the degradative phagolysosome that finally gets rid of the microorganism [7]. In addition, along with a metabolic role in the recycling and degradation of cell components and nutrients, in recent years it has been demonstrated that autophagy actively collaborates with the delivery of the pathogen-containing vacuoles to lysosomes for degradation, in a process specifically known as xenophagy [8], [9].

Once within the host cell, bacteria can take different fates. Pathogens such as *Shigella*, *Listeria* or Group A *Streptococcus* are able to disrupt the phagocytic vacuole and escape into the cytosol, where they manage to replicate and subsequently spread into neighbouring cells [10], [11]. On the other hand, other bacteria, as is the case of *Mycobacterium* or *Salmonella*, can persist inside a phagocytic vacuole delaying or preventing the progression to a phagolysosome, or modifying the features of the lytic compartment. In addition, a variable fraction of the intracellular pathogen population can be targeted by the autophagic system [12], [13]. Free cytosolic bacteria can be wrapped by the nascent autophagosomal vesicle (phagophore) into a double membrane vacuole, the autophagosome, which fuses with lysosomal vesicles, finally forming a degradative autophagolysosome. In the case of membrane-bound bacteria, the compartment can be also sequestered by a phagophore, possibly attracted by signals of membrane damage originated by bacterial effectors, and once more, this structure is fused to lysosomes giving rise to an autolysosome.

While pathogens like *Shigella flexneri* and *Listeria monocytogenes* are able to evade autophagy recognition [14], [15], it was shown that others, like *Brucella abortus*, *Coxiella burnetii* or *Legionella pneumophila,* can replicate in autophagosome-like compartments (reviewed in [8]). Therefore, and in spite of the array of host cell protective strategies, numerous pathogens are able to hijack the cellular defensive machinery at different stages, escaping from, avoiding, delaying or blocking its normal progression, to finally establish an intracellular niche where they can survive and proliferate in the host.

In this work, we explore the invasion process of *S. marcescens* to non-phagocytic cells. We demonstrate that, once inside the cell, *Serratia* is able to inhabit and proliferate inside a large membrane-bound compartment. These vesicles exhibit autophagic-like features as they acquire LC3 and Rab7, markers that have been demonstrated to be recruited throughout the progression of the autophagosome biogenesis in the antibacterial process [16]–[18]. However, we show that the vast majority of the autophagic vacuole population is non-acidic and has no degradative properties, indicating an alteration of the normal delivery to lysosomal compartments. In addition, our results put forth that non-canonical autophagy, which does not involve phosphatidylinositol 3-kinase (PI3K) activity, can be induced by the bacteria before internalization, from outside the target cell. We also show that flagellar expression participates in the early interaction of the bacteria with the host cell and provide evidences indicating that the internalization process engages PI3K activity and requires intravacuolar acidification. These findings shed light on the mechanism that *Serratia* employs to invade and survive inside non-professional phagocytic cells and also reveal that *S. marcescens* constitutes an attractive model organism to further explore the strategies that opportunistic pathogens have evolved to succeed in the host.

## Materials and Methods

### Materials

α-MEM cell culture media, fetal calf serum (FCS), Lipofectamine 2000 and Earlés Balanced Salt Solution (EBSS) were obtained from Invitrogen (Argentina). Antibiotics kanamycin (50 µg/ml), ampicillin (100 µg/ml), chloramphenicol (20 µg/ml), and spectinomycin (100 µ/ml), bafilomycin A1, NH_4_Cl and wortmannin were purchased from Sigma (Argentina).

Rabbit anti-*S. marcescens* polyclonal antibodies were prepared in our laboratory and the secondary goat anti-rabbit IgG (H+L) antibodies conjugated with Cy3 were provided by Zymed Laboratories. Secondary goat anti-rabbit IgG (H+L) antibodies conjugated with Alexa Fluor 647, rabbit anti-GFP polyclonal antibodies, self-quenched red Bodipy dye conjugated to BSA (DQ-BSA), and LysoTracker were purchased from Molecular Probes.

### Bacterial strains and plasmids

The strains and plasmids used in this study are listed in **Table 1** **.** pGFP was obtained by sub-cloning *gfpmut3.1* gene from pGFPmut3.1 (reppMB1 Ap^R^ *gfpmut3.1*, [19]) into pBBR1-MCS2 between *Xho*I and *Hind*III sites. *S. marcescens flhD* was disrupted by integration of the suicide plasmid pKNOCK-Cm^R^ [20]. Briefly, an internal fragment of 317 bp from gen *flhD* was amplified by PCR using primers FlhD-Nter-b42-pknock and FlhD-Cter-rev-Serratia (5′- CGGGATCCTGACATCAATTTGTC-3′ and 5′-TCCAAGCTTCAGGCTCTTTTCTTCG-3′, respectively). The purified PCR product was digested with the *Bam*HI and *Sal*I restriction enzymes and the fragment of 217 bp was ligated into the *Bam*HI and *Sal*I sites of pKNOCK-Cm^R^. The resulting plasmid was introduced into competent *E. coli* SM10(λ_pir_) cells by electroporation and then mobilized into *S. marcescens* RM66262 by conjugation. Insertional mutants were confirmed by PCR analysis. For complementing *S. marcescens flhD*, pBBR1-MCS2 Kn^R^ was digested with *Xho*I and the 5′overhanging end was filled by treatment with Klenow. Finally, it was digested with *Hind*III restriction enzyme. *flhDC* was amplified by PCR using primers FlhD-serratia-Nter and FlhC-CTR (5′-TCGCCCGGGATGGGGAATATGGGTAC-3′ and 5′-TCCAAGCTTCAGGCTCTTTTCTTCG-3′, respectively) and the purified PCR product was digested with the *Sma*I and *Hind*III restriction enzymes. The fragment was ligated into pBBR1-MCS2 Kn^R^ and the resulting plasmid was mobilized into *S. marcescens flhD* by conjugation.

**Table 1:** Strains and plasmid used in this study.

Strains	Relevant phenotype	Source or Reference
***S. marcescens***		
*wild-type*	*S. marcescens* RM66262, clinical isolate	[31]
*flhD*	*S. marcescens* RM66262 *flhD*::pKNOCK, non flagellated, Cm^R^	This work
*phlAB*	*S. marcescens* RM66262 *phlAB*::Cm^R^	[31]
**Plasmids**		
pBBR1-MCS2	Kn^R^, broad range host	[41]
p*flhDC*	pBBR1-MCS2::*flhDC*, Kn^R^	This work
p*phlAB*	pBBR1-MCS2::*phlAB*, Kn^R^	[31]
pGFP	pBBR1-MCS2::*gfpmut3.1*, Kn^R^	This work
pRFP-LC3	*lc3* sub-cloned in the pRFP vector, as described	[33]

The pEGFP-Rab7 plasmid was generously provided by Dr. Bo van Deurs (University of Copenhagen, Copenhagen, Denmark). Genetic manipulations and sequence verifications were performed following previously described protocols [21], [22].

### Bacterial and cell culture

Bacteria were routinely grown in Milleŕs Luria-Bertani (LB) medium supplemented with antibiotics at 30°C, as indicated. Bacterial growth curves performed in LB in the presence or absence of 100 nM bafilomycin A1, 100 nM wortmannin or 10 mM NH_4_Cl were monitored in a 96-microwell plate reader BioTek ELx808, with temperature control (37°C), over a 18 h period. No difference in the growth curves of bacteria incubated in the presence of the inhibitors was observed when compared to bacteria grown in LB medium with no additions.

Chinese Hamster Ovary (CHO) [23], or the derived stably transfected CHO cells over-expressing EGFP-LC3 (CHO-EGFP-LC3), were grown in α-MEM supplemented with 10% FCS at 37°C and 5% CO_2_. HeLa [24], or T24 cells [25], wild-type MEF (Mouse Embryonic Fibroblast) and MEF *Atg5* ^−/−^cells (kindly provided by Dr. Tamotsu Yoshimori, Osaka University, Japan) and the derived cells transiently expressing RFP-LC3 were grown in D-MEM supplemented with 10% FCS in the same conditions as CHO cells. Cells were transfected using Lipofectamine 2000 according to the manufactureŕs instructions.

### Gentamicin protection assay

The gentamicin protection assay was performed as previously described [19] with modifications, as follows. CHO and CHO-GFP-LC3 cells were cultured in 24 wells plates until they reached confluence. *S. marcescens* were grown without shaking, overnight at 30°C. The bacterial cultures were washed once with PBS and an appropriate volume was added to each well to reach MOI 10. Plates were centrifuged 10 min at 1000 rpm and incubated one hour at 37°C and 5% CO_2_. Then, the cells were washed with PBS and subsequently incubated with medium supplemented with 10% FCS and 25 µg/ml gentamicin. Cells were then washed four times with PBS and lysed with 0.05% Triton X-100 at indicated time-points. The resultant supernatants were serially diluted and the CFU on LB agar plates were determined. The results for each experiment are the average of an assay performed in triplicate and independently repeated three times.

To determine cellular damage, lactose dehydrogenase activity (LDH) in the CHO cell culture supernatant was measured with a Wiener Laboratories S.A. (Argentina) LDH kit in accordance with the manufacturer's instructions. Percent LDH release was calculated relative to that of the uninfected control, which was set at 0% LDH release, and that of cells lysed in Triton-X 100 (0.05%) treated cells which was set at 100% LDH release.

### Adherence assay

Bacterial adherence assays were performed as described [26]. Briefly, CHO cells were cultured in 24 wells plates until they reached confluence. *S. marcescens* were grown without shaking, overnight at 30°C. The bacterial cultures were washed once with PBS and the cells were infected (MOI 10). Plates were centrifuged 10 min at 1000 rpm and incubated one hour at 37°C and 5% CO_2_. The cells were then washed four times with PBS and lysed with 0.05% Triton X-100. The resultant supernatants were serially diluted and the CFU on LB agar plates were determined. The results for each experiment are the average of an assay performed in triplicate and independently repeated three times.

### Indirect immunofluorescence and confocal microscopy

Infected cells overexpressing EGFP-Rab7 or EGFP-LC3 were fixed with 0.5 ml of 3% paraformaldehyde solution in PBS for 10 min and permeabilized with 0.1% Triton X-100 solution in PBS for 5 min. Subsequently, cells were incubated with primary polyclonal antibodies against *S. marcescens* (1∶500) and detected by incubation with anti-rabbit Cy3 conjugated secondary antibodies (1∶150) or anti-rabbit Alexa Fluor 647 conjugated secondary antibodies (1∶300). Cells were mounted with SlowFade Antifade reagent in glycerol/PBS (Molecular Probes). Finally, cells were analyzed by confocal microscopy with a Nikon Eclipse TE-2000-E2 and the EZ-C1 3.20 Free Viewer program (Nikon, Japan) or with an Olympus FluoViewTM FV1000 confocal microscope (Olympus, Argentina), and the FV10-ASW (version 01.07.00.16) software, respectively.

### Colocalization of intracellular bacteria with different markers

At different times after infection, cells were fixed for 10 min in 3% paraformaldehyde. Intracellular bacteria were detected by indirect immunofluorescence. At least 200 cells were scored from three independent experiments using confocal microscopy. The percentage of colocalization was calculated as the ratio between the number of vesicles containing bacteria that colocalize with the indicated fluorescently labeled marker and the total number of infected cells. For this purpose, either small vacuoles or large vacuoles filled with bacteria were counted as one vesicle.

### Detection of degradative compartments with chromogenic DQ-BSA

The ability of CHO cells to endocytose and degrade the self-quenched red Bodipy dye conjugated to BSA (DQ-BSA, Molecular Probes) was used to measure degradative lysosomal function. Red DQ-BSA requires enzymatic cleavage in a degradative intracellular compartment to generate a highly fluorescent product which can be monitored by confocal microscopy. CHO-EGFP-LC3 cells were preincubated for four hours at 37°C with DQ-BSA (10 µg/ml in α-MEM supplemented with 10% FCS) to assure that the probe reaches the lysosomal compartment. Subsequently, the cells were infected with *S. marcescens* as described above and four or six hours post-infection the cells were fixed. Intracellular bacteria were detected by indirect immunofluorescence and the cells were analyzed by confocal microscopy. The percentage of colocalization was calculated as the ratio between the number of vesicles containing bacteria that colocalize with the indicated fluorescently labeled markers and the total number of infected cells.

### Detection of acidic compartments with LysoTracker

CHO-EGFP-LC3 cells were infected with *S. marcescens* and, at sixty minutes p.i. the acidic compartments were labeled by incubation of infected cells with 3 µM LysoTracker in α-MEM supplemented with 10% FCS at 37°C. Finally, at four or six hours p.i. cells were fixed and intracellular bacteria were detected by indirect immunofluorescence. Cells were analyzed by confocal microscopy. The percentage of colocalization was calculated as the ratio between the number of vesicles containing bacteria that colocalize with the indicated fluorescently labeled markers and the total number of infected cells.

### Gentamicin protection assay in starvation medium and inhibition by wortmannin

The invasion assays using starvation medium or with the addition of wortmannin (Wn) were performed as the previously described gentamicin protection assay with modifications, as follows. CHO-GFP-LC3 cells were cultured in 24 wells plates until they reached confluence. The cell monolayer was pre-incubated for two hours with medium supplemented with 10% FCS with or without Wn 100 nM or starvation medium EBSS with or without Wn 100 nM. *S. marcescens* cultures were grown without shaking, ON at 30°C. The bacterial cultures were washed once with PBS and an appropriate volume was added to each well to reach MOI 10, and Wn 100 nM was added where indicated. Plates were centrifuged 10 min at 1000 rpm and incubated one hour at 37°C and 5% CO_2_. Then, the cells were washed with PBS, incubated with medium supplemented with 10% FCS and 25 µg/ml gentamicin with the addition of Wn 100 nM where indicated. After 120 or 360 min, cells were washed four times with PBS and lysed with 0.05% Triton X-100. The resultant supernatants were serially diluted and the CFU on LB agar plates were determined.

In another set of experiments, Wn 150 nM was added at 60 min p.i. together with gentamicin. At 210 min p.i. the medium was renewed. After 360 min, cells were washed four times with PBS and lysed with 0.05% Triton X-100. The resultant supernatants were serially diluted and the CFU on LB agar plates were determined. Alternatively, cells were mounted and analyzed by confocal microscopy as described above. The results for each experiment are the average of an assay performed in triplicate and independently repeated three times.

### Gentamicin protection assay with bafilomycin A1 or NH_4_Cl

The invasion assays with the addition of bafilomycin A1 (Baf) or NH_4_Cl were performed as the previously described gentamicin protection assay with modifications, as follows. CHO-GFP-LC3 cells were cultured in 24 wells plates until they reached confluence. *S. marcescens* cultures were grown without shaking, ON at 30°C. The bacterial cultures were washed once with PBS and an appropriate volume was added to each well to reach MOI 10, and 100 nM Baf or 10 mM NH_4_Cl was added. Plates were centrifuged 10 min at 1000 rpm and incubated one hour at 37°C and 5% CO_2_. Then, the cells were washed with PBS, incubated with medium supplemented with 10% FCS and, 25 µg/ml gentamicin with the addition of 100 nM Baf or 10 mM NH_4_Cl. After 120, 240 or 360 min, cells were washed four times with PBS and lysed with 0.05% Triton X-100. The resultant supernatants were serially diluted and the CFU on LB agar plates were determined. Alternatively, cells were mounted and analyzed by confocal microscopy as described above. The results for each experiment are the average of an assay performed in triplicate and independently repeated three times.

### Immunodetection of EGFP-LC3

CHO-EGFP-LC3 cells from the invasion assays where lysed with SDS-PAGE sample buffer. Protein samples from the total cell lysate were run on a 10% polyacrylamide gel (30 µg were loaded per lane) and transferred to Hybond-ECL nitrocellulose membranes (GE Healthcare). The membranes were blocked for 2 h with 5% non-fat milk, 0.1% Tris-Buffer Saline (TBS), and washed twice in TBS for 10 min. Then the blots were incubated with primary rabbit anti-GFP polyclonal antibodies, washed twice in TBS and finally incubated with peroxidase-conjugated secondary antibody (GE Healthcare). The blots were developed using an enhanced chemiluminescence detection kit (ECL-Plus) from GE Healthcare. In parallel, the same procedure was carried out to detect the protein α-tubulin, used as loading control. Quantification of individual bands by densitometry and histograms were performed using the Image J Program.

### Gentamicin protection assay in bacteriostatic conditions

CHO-EGFP-LC3 cells were cultured in 24 wells plates until they reached confluence. Overnight *S. marcescens* cultures were washed once with PBS and incubated one hour at 30°C in LB supplemented with 20 or 100 µg/ml chloramphenicol. Subsequently, cells were infected (MOI 10) and incubated one hour in α-MEM supplemented with 10% FCS and 20 or 100 µg/ml chloramphenicol. Then, the cells were washed with PBS and medium supplemented with 10% FCS and 25 µg/ml gentamicin was added. After two hours, cells were fixed and intracellular bacteria were detected by indirect immunofluorescence. At least 300 cells were scored from three independent experiments using confocal microscopy. The percentages of invasion and colocalization with LC3 were calculated as the ratio between the total number of infected cells and the total number of cells, or the number of internalized bacteria that colocalize with LC3 and the total number of cells, respectively.

For the assays using heat-killed bacteria, overnight *S. marcescens* cultures were washed once with PBS and incubated 20 minutes at 65°C. Loss of bacterial viability was verified by determination of CFUs in LB-agar plates. Subsequently, CHO-EGFP-LC3 cells were infected (MOI 10) and incubated one hour in α-MEM supplemented with 10% FCS. Then, an identical protocol to the one described for live bacteria was carried out to determine intracellular CFUs. The results for each experiment are the average of an assay performed in triplicate and independently repeated three times.

### Statistical analysis

Statistical analysis was performed using one-way ANOVA and Holm-Sidak test with an overall significance level  = 0.05. Asterisks in the plots denote the values among the treatment groups in which a statistical significant difference was determined.

## Results

### Survival and replication of *S. marcescens* inside epithelial cells

**Figure 1:**
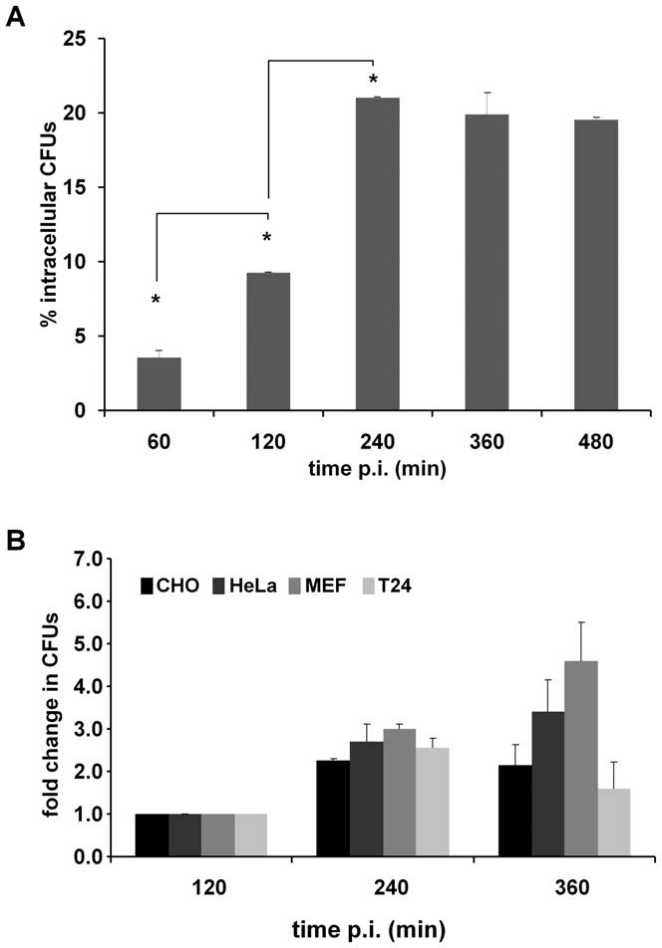
*S. marcescens* invades and replicates inside non-phagocytic cells. **A)** CHO cells or **B)** CHO, HeLa, MEF *Atg5* ^+/+^ and T24 cells were infected for 60 min with wild-type *S. marcescens* and then extracellular bacteria were eliminated with gentamicin. At the indicated times, cells were washed with PBS and lysed with 0.05% Triton X-100. The CFUs were determined on LB agar plates, percentages were calculated relative to CFUs in the inoculum. In the plot shown in **(B)** fold change in CFUs were calculated relative to the values obtained at 120 min p.i. The average ± S.D. for two independent experiments is shown (* p<0.001).

**Figure 2:**
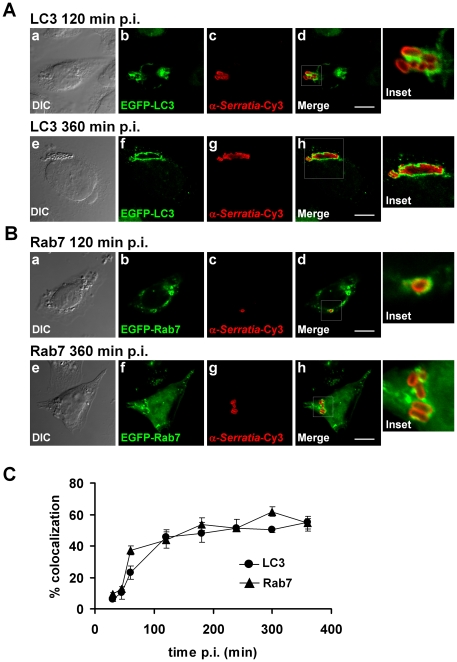
LC3 and Rab7 are recruited to the *S. marcescens*-containing vacuole. **A**) CHO-EGFP-LC3 cells (green fluorescence) or **B**) CHO cells transfected with EGFP-Rab7 (green fluorescence), were infected with wild-type *S. marcescens* and cells were fixed at 120 min p.i (a-d) or 360 minutes p.i (e-h). Bacteria were detected with antibodies anti-*Serratia* coupled with a secondary antibody labeled with Cy3 (red fluorescence). Representative confocal Z-slices are shown. Inset images show higher magnification of the boxed areas in merged images. Bars: 10 µm. **C)** CHO-EGFP-LC3 cells or CHO cells transfected with pEGFP-Rab7 were infected with wild-type *S. marcescens*. Cells were fixed at the indicated time points and bacteria were detected with antibodies anti-*Serratia* coupled with a secondary antibody labeled with Cy3. The percentage of colocalization of EGFP-LC3 (•) or EGFP-Rab7 (▴) with bacteria was determined by fluorescence microscopy. At least 200 infected cells were counted for each time point. The average ± S.D. for three independent experiments is shown.

Upon infection of CHO epithelial cells, intracellular cumuli of labeled *Serratia* inside CHO cells were observed by phase contrast and by fluorescence microscopy suggesting that bacteria were enclosed within vacuole-like compartments. The score of the CFUs recovered intracellularly along time showed that *Serratia* was able not only to survive but also to replicate inside the cells with an estimated 42.8 min doubling time (49 min was the doubling time determined for *S. marcescens* grown in LB, at 37°C, without agitation), leading to an increase of up to 6-fold in the intracellular counts at 240 min p.i., with no significant decay up to 480 min p.i. (Fig. 1A, representative images of CHO invaded cells at different times p.i. are shown in Fig. 2 and subsequent). Beyond this point, if gentamicin action was eliminated, although CHO cellular integrity was maintained at early times as indicated by monitoring LDH release (see Materials and Methods for details), bacteria slowly started to appear in the extracellular space indicating that they could somehow exit the CHO cells without causing detectable cell damage. If these few extracellular bacteria were not eliminated by restoring gentamicin action, their rapid proliferation caused bacterial numbers that provoked the rounding and detachment of the CHO cells, as well as release of LDH, indicative of cellular damage (equivalent to the results obtained if a MOI higher than 20 was employed in the infection assay, not shown). Further experiments are under way in our laboratory to examine the fate of *Serratia* and the mechanism that underlies cellular escape at late times p.i.

An identical invasion (gentamicin protection) assay to the one performed with CHO cells was also carried out using HeLa, MEF or T24 epithelial cell lines. Intracellular CFUs were determined at 120, 240 and 360 min p.i. and CFUs fold change relative to the value obtained at 120 min p.i was plotted. As shown in Fig. 1B, *Serratia* invasion and intracellular replication was sustained by the four cell lines assayed. Upon 360 min p.i. T24 cells microscopically showed signals of cellular damage (rounding and detaching, not shown) that correlated with the intracellular CFUs decrease observed at this time point. Together, these results indicate that the capacity of *S. marcescens* to invade and proliferate intracellularly is not restricted to one specific epithelial cell type.

### The autophagy system targets the *Serratia*-containing vacuole

To characterize the intracellular structures that harbor *Serratia*, that we will hereafter call *Serratia*-containing vacuoles (SeCVs), and address whether they interact with the autophagy pathway, we used a stably transfected CHO cell line expressing the EGFP-LC3 fusion protein. We previously verified that the *Serratia* infection time-course using EGFP-LC3-expressing CHO cells was identical to the one determined for non-transfected CHO cells (not shown). Fig. 2A shows representative images obtained from colocalization assays at 120 or 360 min p.i.; in this assay SeCVs were observed decorated by EGFP-LC3. Colocalization was calculated as the ratio between the number of SeCVs that colocalize with the fluorescently labeled marker and the total number of infected cells. After bacterial entry, a gradual increase in *Serratia* colocalization with EGFP-LC3 was detected, reaching 47.9±2.9% colocalization 180 min p.i., with no detectable decay up to 360 min p.i. (Fig. 2C).

Maturation of autophagosomes is a multi-step process that involves fusion events with vesicles originating from the endo/lysosomal compartment. Rab7 is a GTPase involved in coordinating tethering/docking of vesicles to late endosomes/lysosomes, either in homotypic or heterotypic fusion events [27]. In this context, it has been shown that a functional Rab7 GTPase is targeted to autophagic vacuoles, being its acquisition involved in the maturation of autophagosomes engaged in bacterial elimination [16]–[18]. To further characterize the autophagic-like vacuoles associated with internalized *Serratia*, we tracked bacterial colocalization with Rab7 following infection of transiently transfected CHO cells overexpressing EGFP-Rab7. Representative images of *Serratia* colocalization with Rab7 acquired at 120 min and 360 min p.i. are shown in Fig. 2B. Rab7 can be observed decorating the SeCVs. Colocalization with Rab7 increased after bacterial internalization reaching 53.7 ± 2.0% at 180 min p.i., a score that was in average sustained up to 360 min p.i. (Fig. 2C). It is worth highlighting that a comparable acquisition kinetic pattern and percentages of colocalization were obtained for LC3 and Rab7. This observation strongly suggests that both markers are recruited to the *Serratia*-containing compartment early on after bacterial internalization and remain associated throughout its progression to become a large crowded vacuole.

**Figure 3:**
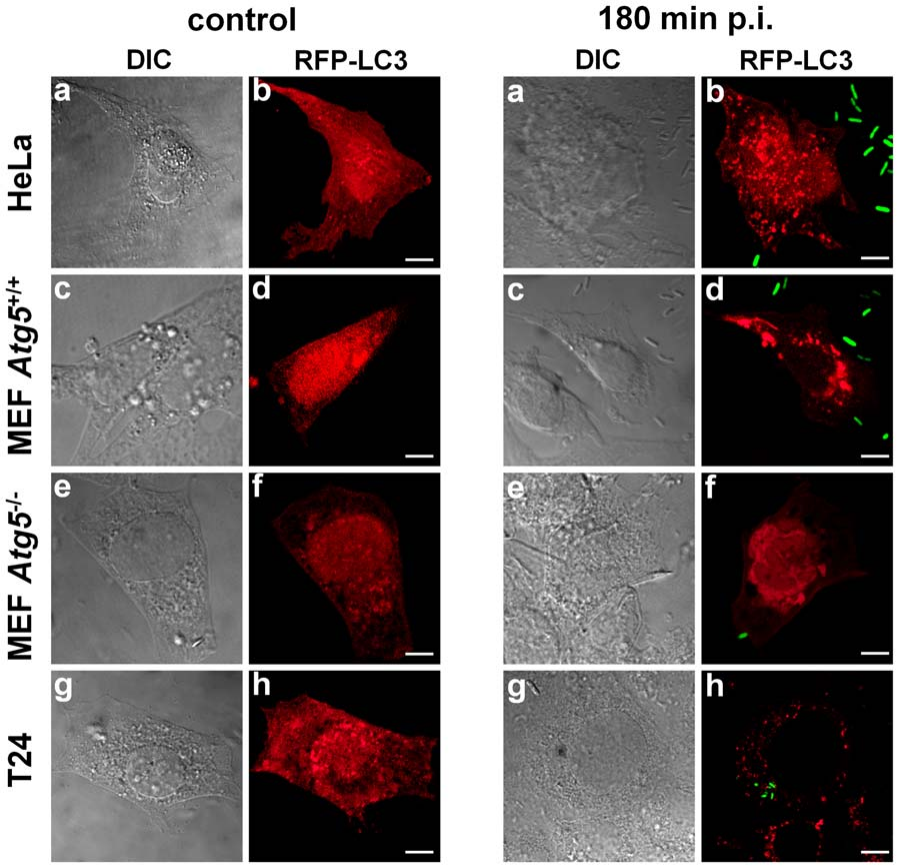
*Serratia* is able to induce autophagy in different epithelial cell lines. HeLa, MEFs *Atg5* ^+/+^, MEF *Atg5* ^−/−^ and T24 cells were transiently transfected with pRFP-LC3 (left panels, red fluorescence) and invaded with wild-type *S. marcescens* transformed with pGFP (right panels, green fluorescence). Cells were fixed at 180 min p.i. and visualized by confocal laser microscopy. Images are representative of at least 300 infected cells monitored in two independent assays. Bars: 10 µm.

In addition, the induction of the autophagic process evidenced microscopically as the punctate LC3 pattern was also observed when HeLa, MEF or T24 cells transiently expressing ERFP-LC3 were challenged with *Serratia* following the same infection protocol as used for CHO-EGFP-LC3 cells without addition of gentamicin (Fig. 3, panels a–d and g–h). This result demonstrates that the autophagic induction in response to *Serratia* invasion is not limited to CHO cells.

### SeCVs population is predominantly composed of non-acidic, non-degradative autophagic compartments

During the course of its maturation process, a canonical autophagocytic vacuole fuses with compartments from the endo/lysosomal pathway, undergoing acidification and the acquisition of hydrolytic enzymes that become active in this environment. Consequently, we analyzed whether the LC3-decorated SeCVs underwent acidification. For this purpose, Lysotracker, a fluorescent marker that labels acidic compartments, was added to the cell cultures 60 min p.i.

**Figure 4:**
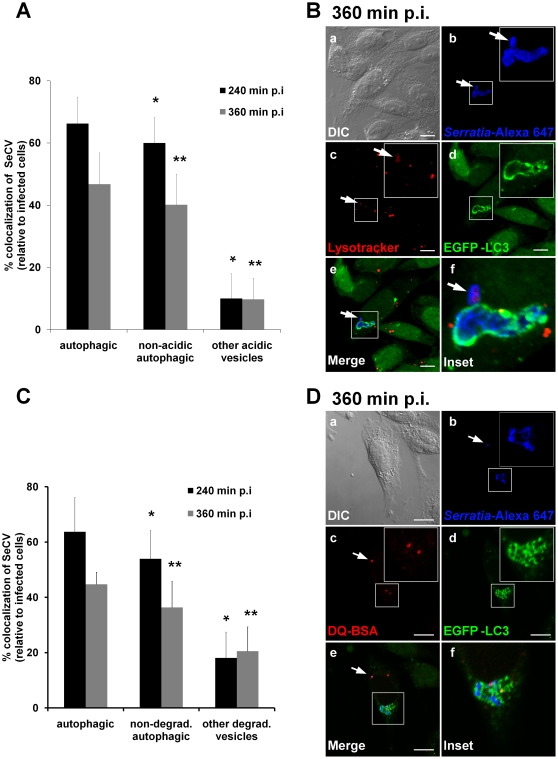
The *S. marcescens*-containing vacuoles are predominantly non acidic and non degradative compartments. **A**) CHO-EGFP-LC3 cells were infected with wild-type *S. marcescens.* After 60 min p.i, cells were incubated with 3 µM of LysoTracker to label acidic compartments. Cells were fixed at 240 or 360 min p.i. and intracellular bacteria were detected with antibodies anti-*Serratia* coupled with a secondary antibody labeled with Alexa Fluor 647. The percentages of colocalization of bacteria with EGFP-LC3 and/or with Lysotracker were determined by fluorescence microscopy. At least 300 infected cells were counted for each condition. The average ± S.D. is shown for two independent experiments, *and **p<0.001. **B**) Representative confocal laser captured images of CHO-EGFP-LC3 cells (green fluorescence, d) infected with *S. marcescens* (blue fluorescence, b) and incubated with Lysotracker (red fluorescence, c) at 360 min p.i. are shown. Inset image (f) shows higher magnification of the boxed area in the merged image (e), and highlights a non-acidic, autophagic SeCV. Arrows point at acidic vesicles. Bars: 10 µm. **C)** CHO-EGFP-LC3 cells were pre-incubated with 10 µg/ml of DQ-BSA for 4 hours to label degradative compartments. Subsequently, cells were infected with wild-type *S. marcescens* and fixed at 240 or 360 min p.i. Intracellular bacteria were detected with antibodies anti-*Serratia* coupled with a secondary antibody labeled with Alexa Fluor 647. The percentages of colocalization of bacteria with EGFP-LC3 and/or with DQ-BSA were determined by fluorescence microscopy. At least 300 infected cells were counted for each condition. The average ± S.D. is shown for two independent experiments, *and **p<0.001. **D)** Representative confocal laser captured images of CHO-EGFP-LC3 cells (green fluorescence, d) pre-incubated with DQ-BSA (red fluorescence,c) and infected with *S. marcescens* (blue fluorescence, b) at 360 min p.i. are shown. Inset image (f) shows higher magnification of the boxed area in the merged image (e) and highlights a non-degradative, autophagic SeCV. Arrows point at degradative vesicles. Bars: 10 µm.

At 240 min p.i. co-localization of SeCVs and LC3 was estimated in 66.2%. However, 60% were LC3-decorated but did not co-localize with Lysotracker, indicating that they were non-acidic autophagic compartments. An average of 10% were found to be SeCVs that were labeled by the acidotropic marker, while they did not colocalize with LC3 (quantitation of the *Serratia*-containing vesicles colocalization with EGFP-LC3 relative to total infected cells and Lysotracker assay at 240 and 360 min p.i. is given in Fig. 4A; representative captured images from 360 min p.i. of the colocalization assay, indicating both acidic –arrows- and non-acidic vacuoles –inset-, are shown in Fig. 4B).

We simultaneously evaluated if LC3-labeled SeCVs displayed features of degradative compartments. With this aim, DQ-BSA was preloaded into GFP-LC3-expressing CHO cells 4 hours previous to infection. DQ-BSA is a self-quenched fluorophore that emits red fluorescence upon proteolytic digestion, useful to monitor degradative vacuoles. In this assay, at 240 min p.i., co-localization of SeCVs and LC3 was estimated in 66.6%., while 53.9% SeCV were LC3-labeled vesicles and did not show co-localization with DQ-BSA. 18.1% SeCVs were labeled by the degradative marker but not by LC3 (quantitation of the *Serratia*-containing vesicles colocalization relative to total infected cells with EGFP-LC3 and DQ-BSA assay at 240 and 360 min p.i. is given in Fig. 4C, representative captured images from 360 min p.i., indicating both degradative -arrows- and non-degradative -inset- vacuoles are shown in Fig. 4D). As shown in Figs. 4A and 4C, a similar distribution of vesicles was also determined at 360 min p.i. Collectively, these results clearly indicate that at late times post-infection (i.e. after 4 h p.i.), a predominant population of the internalized *Serratia* is contained in autophagic-like non-degradative and non-acidic compartments, while a minor proportion of total internalized bacteria is delivered to degradative, acidic vesicles (i.e. lysosomal compartments).

**Figure 5:**
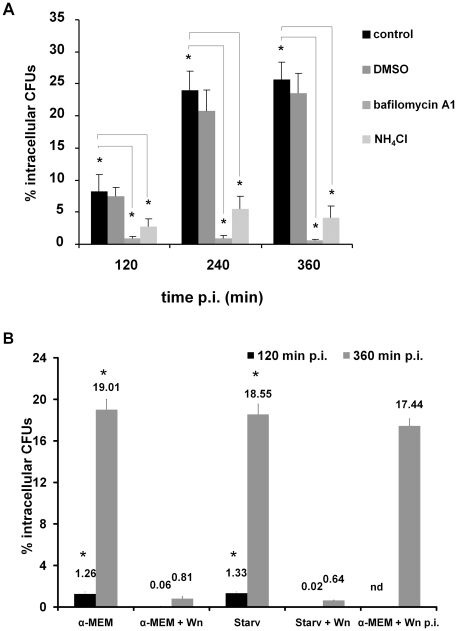
Effect of autophagy inhibitors on *Serratia* invasion. **A) Inhibition or neutralization of vacuolar acidification on** ***Serratia*** **invasion.** CHO-EGFP-LC3 cells were maintained in α-MEM, incubated with DMSO 1% (which equals the final concentration of DMSO used as vehicle for bafilomycin A1), 100 nM bafilomycin A1 or 10 mM NH_4_Cl, as indicated in Materials and Methods. Cells were infected with wild-type *S. marcescens* following the gentamicin protection assay procedure. Invaded cells were lysed with 0.05% Triton X-100 and CFUs were determined on LB agar plates at 120, 240 or 360 min p.i. Percentages were calculated relative to CFUs in the inoculum. The average ± S.D. is shown for three independent experiments, *p<0.001. **B) Modulation of autophagy on *Serratia* invasion.** CHO-EGFP-LC3 cells were maintained in α-MEM, or pre-treated in EBSS starvation medium (Starv). 100 nM Wortmannin (Wn) was added 2 h previous to the infection assay (α-MEM + Wn; or Starv + Wn) or at 60 min p.i. (α-MEM + Wn p.i). Cells were infected with wild-type *S. marcescens* following the gentamicin protection assay procedure. Invaded cells were lysed with 0.05% Triton X-100 and the CFUs were determined on LB agar plates at 120 or 360 min p.i. The average ± S.D. is shown for three independent experiments (% intracellular CFUs are indicated above each bar in the graph; nd: not determined, *p<0.001).

**Figure 6:**
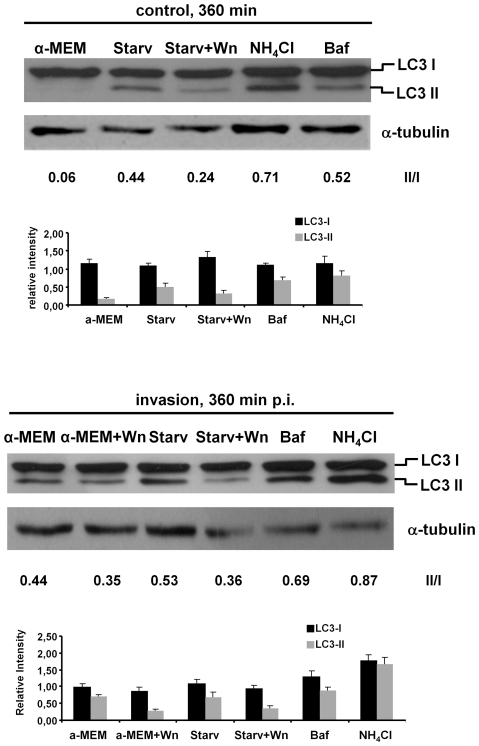
Analysis of LC3-I/LC3-II conversion. CHO-EGFP-LC3 cell monolayers subjected to the invasion assays as described in Figure 5 were harvested and resuspended in SDS-PAGE sample buffer. The samples were resolved by SDS-PAGE (30 µg of total protein was loaded per lane), transferred to nitrocellulose and detected with anti-GFP polyclonal antibodies (“invasion”, lower panel blots). Control, non-invaded CHO-EGFP-LC3 cells, that were subjected to the same treatments were also analyzed (“control”, upper panel blots). The positions of LC3-I and LC3-II bands are indicated. Protein loading was controlled according to the α-tubulin content (the blots detected with antibodies anti-α-tubulin are shown). Blots are representative of three independent experiments. The bands intensity was quantified with the ImageJ Programe, and the LC3II/LC3I (II/I) was calculated for the immunodetection experiment shown in the figure. The histogram represents the average ± S.D. of the relative intensities of LC3-I or LC3-II *versus* α-tubulin of three independent experiments.

**Figure 7:**
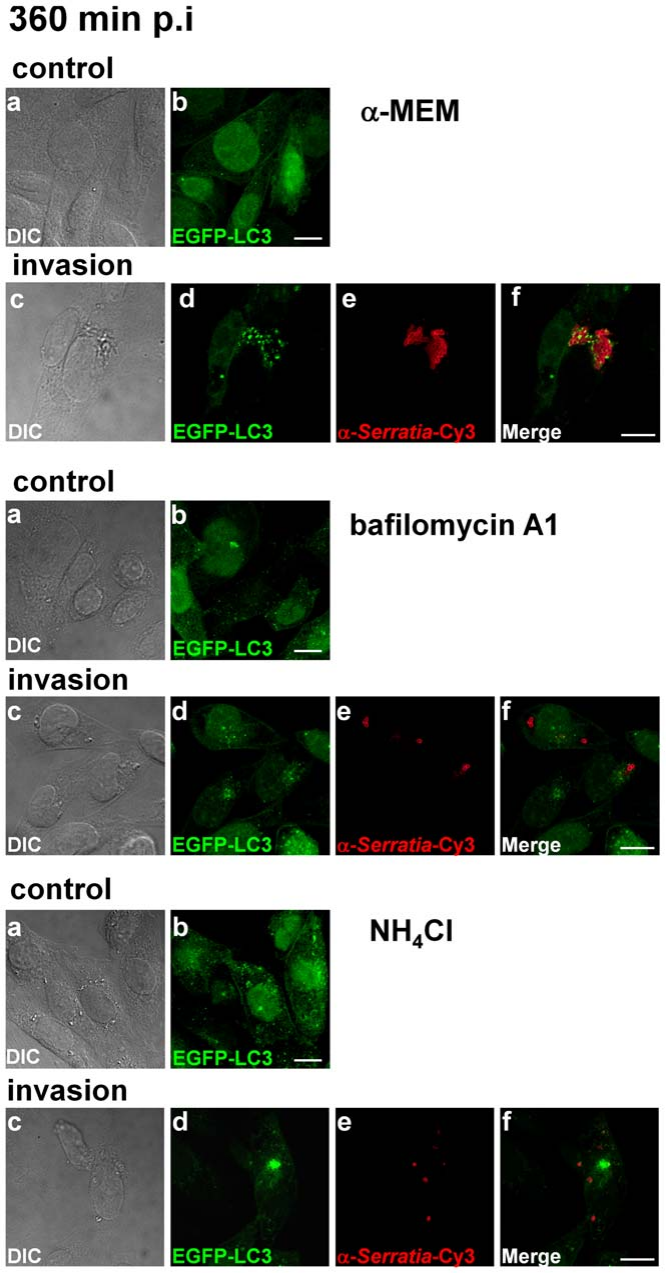
Confocal microscopy analysis of the effect of altering vacuolar acidification on *Serratia* invasion. CHO-EGFP-LC3 cells subjected to the invasion assays as described in Fig. 5A were visualized by confocal laser microscopy. a and b panels show representative images of control, non-invaded cells and c-f panels depict invaded cells incubated with α-MEM, α-MEM +100 nM Baf or α-MEM +10 mM NH_4_Cl, as indicated. Bacteria were detected with antibodies anti-*Serratia* coupled with a secondary antibody labeled with Cy3. Representative DIC (c), EGFP-LC3 green fluorescence (d), red fluorescence-labeled bacteria (e) and merged images (f) are shown. At least 300 cells were inspected for each condition in two independent experiments. Bars: 10 µm.

Next, the invasion assay was carried out in the presence of bafilomycin A1 or NH_4_Cl to block the vacuolar type-H^+^-ATPase action or to neutralize vacuolar acidification, respectively. The induction of autophagy by *Serratia* was examined by the conversion of LC3-I to the lipidated LC3-II form (Fig. 6, compare the LC3-II/LC3-I ratio, 0.06 from control cells and 0.44 from invaded cells with no inhibitor added, which is the result from autophagy induction by *Serratia* and LC3 turn-over). In the presence of bafilomycin A1, the accumulation of LC3-II due to the blockage in protein turn-over was evident (Fig. 6, LC3-II/LC3-I ratio  = 0.71 for NH_4_Cl and  = 0.52 for bafilomycin compared to 0.06 in non-treated control cells). However, in the invasion assay, the LC3-II/LC3-I ratios were similar to the values obtained by non-invaded cells (Fig. 6, compare NH_4_Cl or Baf in control and invasion assays). Besides, the effect of the inhibitors on the intracellular survival of *Serratia* contrasted to the expected outcome that would predict an increase in intracellular CFUs due to the blockage in the delivery of vacuolar compartments to the lysosomal ones. Intracellular recovered CFUs were highly diminished as early as 120 min p.i. (0.91% and 2.71% intracellular CFUs in bafilomycin A1 or NH_4_Cl treated cells, *versus* 8.26% for control cells) and remained without significant changes at 240 or 360 min p.i., when compared to non-treated cells in which *Serratia* proliferated intracellularly (Fig. 5A). As shown in Fig. 7, the punctate LC3 pattern microscopically observed at 360 min p.i. in either invaded or non-invaded cells treated with the inhibitors was also indicative of an effective blockage in LC3 protein turn-over. Scarce SeCVs containing only few bacteria could be detected in treated invaded cells when compared with non-treated cells, in accordance to the low % of intracellular CFUs recovered (Fig.5A, and see Fig.7). This result explains the similarity between the LC3 ratio values obtained for non-invaded and invaded cells treated with the inhibitors: because both compounds inhibit early invasion events, at 360 min p.i. gentamicin action has already eliminated extracellular bacteria giving no time for the extracellular induction of autophagy to develop. Neither bafilomycin A1 or NH_4_Cl, added to the culture media at the final concentrations used in the assay, affected the growth or showed toxic effects on *Serratia* (see Materials and Methods, data not shown). Taking into account that the two different procedures used to prevent intraluminal vacuolar acidification rendered a severe reduction in intracellular CFUs as early as 120 min p.i., it is tempting to speculate that a low pH ambient might be required for early invasion steps.

### *Serratia* promotes a non-canonical autophagic pathway

**Figure 8:**
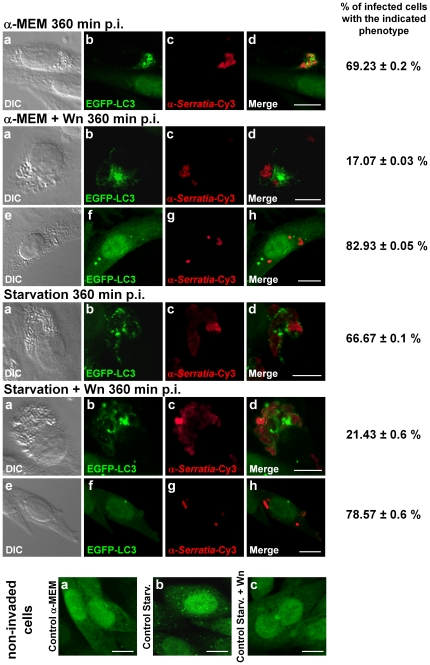
Confocal microscopy analysis of the induction or inhibition of autophagy on *Serratia* invasion in CHO cells. CHO-EGFP-LC3 cells subjected to the invasion assays as described in Fig. 5B were visualized by confocal laser microscopy. Bacteria were detected with antibodies anti-*Serratia* coupled with a secondary antibody labeled with Cy3. Representative DIC (a and e), EGFP-LC3 green fluorescence (b and f), red fluorescence labeled bacteria (c and g) and merged fluorescence images (d and h) are shown. In the assays where wortmannin was used, representative images from both the scarce SeCV observed (a-d) and from barely invaded cell fields (e-h) are shown. The lower panel shows representative images of control, non-invaded cells incubated with α-MEM, in starvation medium or in starvation medium + Wn (green EGFP-LC3 fluorescence, a, b and c, respectively). Bars: 10 µm. The percentage of invaded cells displaying the phenotype shown in the images in the corresponding row is indicated to the right. At least 200 cells were counted for each condition; the media ± S.D. is shown for two independent experiments.

In light of the previous results, we examined whether induction of autophagy by cell starvation would enhance *Serratia* invasion process. We also analyzed the effect of the addition of one of the best-characterized autophagy inhibitors, wortmannin. When we induced autophagy by starving the cells in EBSS medium (see Materials and Methods for details), no increase in intracellular CFUs was detected either at 120 or 360 min p.i. when compared to non-starved cells (Fig. 5B, αMEM and Starv). On the other hand, when wortmannin was used to block autophagy by pre-treatment of the non-starved or starved cells with the inhibitor, intracellular CFUs were drastically diminished in comparison to non-treated cells, both at 120 and 360 min p.i. (Fig. 5B, αMEM + Wn and Starv + Wn). Microscopical inspection of the CHO cells assayed revealed that, when wortmannin was used, the scarce intracellular compartments observed harboring *Serratia* were packed with bacteria and decorated with LC3, exhibiting identical phenotype to the SeCVs observed in the profusely invaded, non-treated cells (Fig. 8, compare α-MEM, panels a–d to α-MEM + Wn, panels a–h, the percentage of CHO cells invaded with *Serratia* that displayed each phenotype is indicated to the right of the images). Representative starved invaded cells are shown either when cells were treated with or without wortmannin (Fig. 8, Starvation, panels a–d, and Starvation + Wn, panels a–h, respectively). It is worth pointing out that the control assay showing the inhibition of autophagy induction by wortmannin pre-treatment in starved non-invaded cells corroborated the expected action of the inhibitor examined microscopically and by immunodetection of LC3 conversion (Fig. 6, compare control assay, α-MEM, Starv and Starv+Wn lanes, and Fig. 8, non-invaded cells, compare panels a–c). These results indicate that treatment with the inhibitor diminished the invasion process, although it was not able to prevent the induction of the autophagic process promoted by *Serratia* as well as the formation of large LC3-decorated SeCV in the few invaded cells observed.

Wortmannin suppresses canonical autophagy by its phosphatidylinositol 3-kinase (PI3K) inhibitory activity [9], therefore, we wondered whether this inhibitor was somehow preventing *Serratia* entry into the CHO cells. To examine this possibility, we added wortmannin after allowing internalization of *Serratia* into CHO cells (60 min p.i.); no detectable difference was obtained in the invasion percentage at 360 min p.i. with the one obtained in non-treated cells (to avoid inactivation of the inhibitor through the assay, fresh medium with wortmannin was replenished at 210 min p.i., Fig.5B, αMEM + Wn p.i.). In accordance with these results, the LC3-II band was detected in all *Serratia* invaded cells irrespective of the treatment applied (i.e. in presence or absence of wortmannin, in either starved or non starved cells; Fig. 6). A lower LC3-II/LC3-I ratio was detected in those lanes that correspond to wortmannin treatment when compared to those from non-treated, invaded cells, reflecting the reduced number of LC3–recruiting SeCVs (Fig. 6, and see also Fig. 5B and Fig. 8). Together, these results show that wortmannin is unable to inhibit the autophagic process triggered by *Serratia*, although it can hinder the bacterial internalization process.

**Figure 9:**
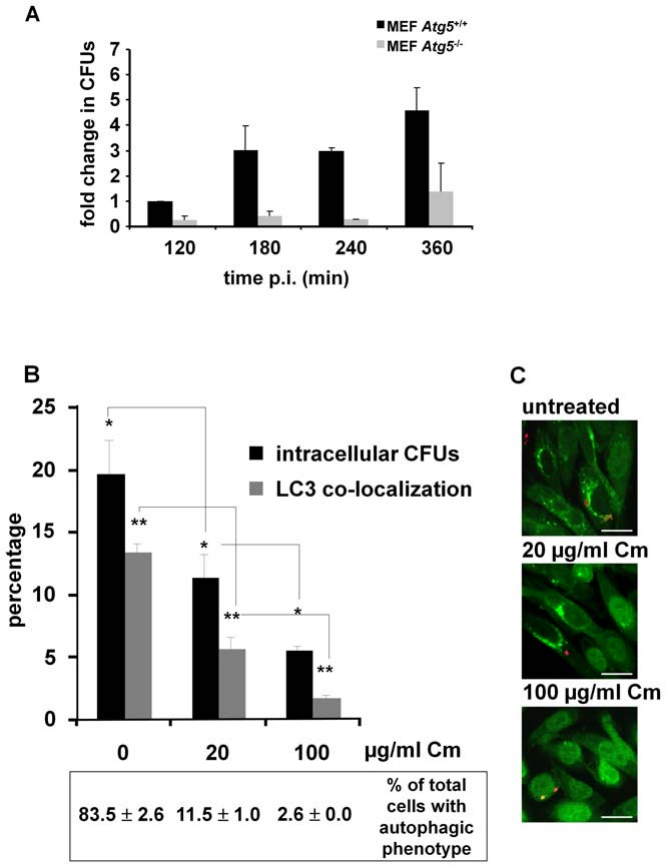
The autophagic process triggered by *Serratia*. **A) Autophagy triggered by** ***Serratia*** **requires Atg5**. MEF *Atg5* ^+/+^ and MEF *Atg5* ^−/−^ cells were infected for 60 min with wild-type *S. marcescenes*. Thereafter, extracellular bacteria were eliminated with gentamicin. At the indicated times, cells were washed with PBS and lysed with 0.05% Triton X-100. The CFUs on LB agar plates were determined. Units were calculated relative to CFU recovered from MEF *Atg5* ^+/+^ cells at 120 min p.i. The media ± S.D. is shown for three independent experiments. **B)** ***S. marcescens*** **actively induces autophagy from the extracellular media.** Overnight *S. marcescens* cultures were incubated 60 min in LB without antibiotic or supplemented with 20 or 100 µg/ml chloramphenicol (bacteriostatic condition). Subsequently, CHO-EGFP-LC3 cells were infected with bacteria and then extracellular bacteria were eliminated with gentamicin (bacteriostatic treatment was maintained throughout the invasion assay). At 180 min p.i, cells were fixed and bacteria were detected with antibodies anti-*Serratia* coupled with a secondary antibody labeled with Cy3. Finally, the percentage of colocalization of vesicles containing *Serratia* with EGFP-LC3, relative to total infected cells, was determined by fluorescence microscopy. The lower panel shows the percentage of cells, infected and non-infected, with an autophagic phenotype. At least 300 infected cells were counted for each condition. The average ± S.D. is shown for three independent experiments, * and ** p<0.001. **C)** CHO-EGFP-LC3 cells (green fluorescence) infected with *S. marcescens* (red fluorescence) untreated (upper panel), treated with 20 µg/ml chloramphenicol (middle panel) or treated with 100 µg/ml chloramphenicol (lower panel) are shown. Bars: 10 µm.

Atg5 (autophagy related factor 5) is essential for the early stages of canonical autophagosome formation, previous to the recruitment of LC3. To further explore the autophagic cascade activated by *Serratia*, wild-type (MEF *Atg5* ^+/+^) and *Atg5* knock-out (MEF *Atg5^−^* ^/−^) cells were used for the bacterial infection assays. The autophagic phenotype was observed in wild-type RFP-LC3-MEF cells, while no punctate pattern was detected in RFP-LC3-MEF *Atg5^−^* ^/−^ cells challenged with *Serratia* (see Fig. 3, panels c–f). In addition, whereas in *Atg5* ^+/+^ cells bacteria augmented its intracellular population in the 120–360 min lapse p.i., no changes were observed for the CFUs recovered at this time period from the knock-out cells (Fig. 9A). In sum, these results indicate the involvement of Atg5, but not of PI3K, in the autophagic route induced by *Serratia*, and suggest that the induction of a non-canonical autophagic pathway is required for the development of the invasion process in epithelial cells.

### Autophagy is activated by a bacterial factor from outside the host cell

We next analyzed whether active bacterial protein synthesis is involved in the invasion process. With this aim, we halted *de novo* protein synthesis by the addition of chloramphenicol (Cm) to the bacterial inoculum, maintaining the action of the bacteriostatic agent throughout the invasion assay. We verified that Cm has bacteriostatic but not bactericidal action at the concentrations and time-lapses used in the assay (determined by recovered CFUs counted before and after Cm treatment of the bacterial cultures, data not shown). The treatment with increasing concentrations of the antibiotic drastically reduced *Serratia* intracellular CFUs (4-fold reduction when 100 µg/ml Cm were used), as well as colocalization of the bacteria with EGFP-LC3 (7-fold decrease when 100 µg/ml Cm were used) when compared with non-treated bacteria (Figs. 9B and 9C, invasion and LC3 co-localization percentages were calculated relative to total scored cells). Remarkably, when we scored the total amount of cells that showed LC3 puncta (Fig. 9B, lower inset) it became evident that not only invaded but also non-invaded cells displayed this autophagic phenotype (also compare phenotypes of invaded to non-invaded cells, Fig. 9C, untreated cells). In addition, when the assay was carried out using heat-killed bacteria (as detailed in Materials and Methods) no differences from non-invaded cells were detected in the intracellular CFUs or in the autophagic phenotype (not shown). These results indicate that metabolically active bacteria are required for an efficient invasion process and for autophagy induction. Additionally, they strongly suggest that the autophagic response can be promoted by *Serratia* by a heat-labile factor from outside the host cell.

### Flagellar expression is involved in the early steps of bacterial interaction with the host cell

In a variety of bacterial pathogens such as *Burkholderia*, *Aeromonas* or enterotoxigenic *E. coli* the flagellum was shown to be involved in initial adherence to the target cell [28]–[30]. In search for bacterial factors that could be involved in the first steps of *Serratia* extracellular interaction with the epithelial cells, we analyzed a *Serratia flhD* mutant strain, impeded in flagellum biogenesis.

**Figure 10:**
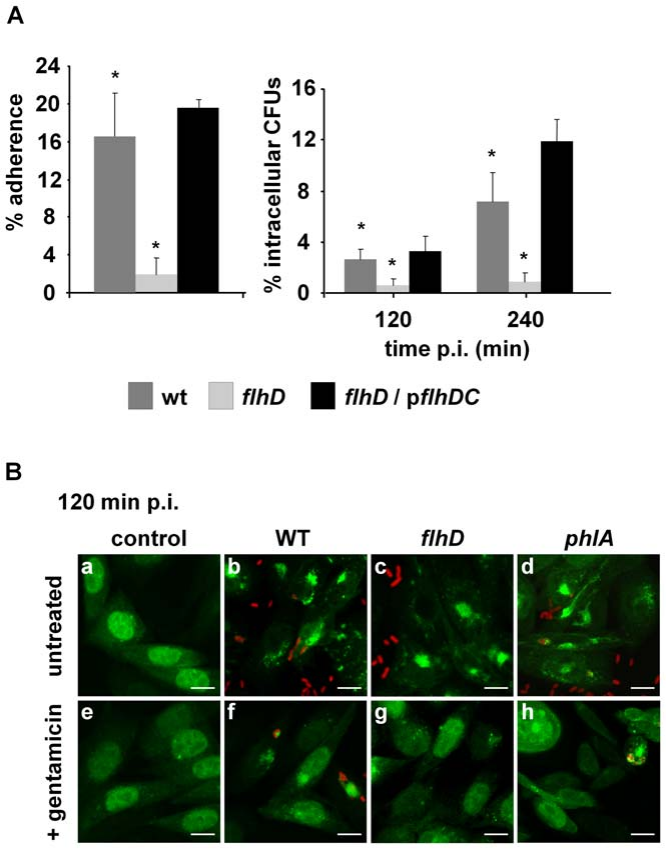
*S. marcescens* flagellum expression is involved in adhesion to CHO cells. **A)** CHO cells were infected for 60 min with *S. marcescens* wild-type, *flhD* and *flhD/*p*flhD* strains (dark gray, light gray or black bars, respectively). Cells were washed with PBS and lysed with 0.05% Triton X-100 (left graphic); or incubated 120 o 240 min p.i with medium supplemented with gentamicin to eliminate extracellular bacteria. Finally, cells were lysed with 0.05% Triton X-100 (right graphic) and the CFUs were determined on LB agar plates. Adherence and intracellular CFUs percentages were calculated relative to initial inoculums. The average ± S.D. is shown for four independent experiments, * p<0.001. **B)** CHO-EGFP-LC3 cells (green fluorescence) were infected with *S. marcescens* wild-type (b and f), *flhD* (c and g) and *phlA* (d and h) strains. Non infected cells are shown as control (a and e). Then, cells were not treated (a-d) or treated with gentamicin (e-h) for 120 min. Cells were fixed and bacteria were detected with antibodies anti-*Serratia* coupled with a secondary antibody labeled with Cy3 (red fluorescence). Representative confocal Z-slices are shown. Bars: 10 µm.

In *S. marcescens* the flagellum biogenesis depends on a regulatory cascade mastered by the FlhD_2_C_2_ transcriptional factor. FlhD_2_C_2_-modulated cascade also couples flagellar biogenesis to the expression of PhlA, a phospholipase of unknown function that is exported through the flagellar secretome [31]. As shown in Fig. 10A, a *flhD* mutant strain was severely impaired both in adherence (8-fold reduction) and in invasion (4-fold or 8-fold reduction, estimated at 120 or 240 min p.i., respectively) when compared to the wild-type strain. Both adhesion and invasion were restored to wild-type levels when the *flhD* mutant strain was complemented by the expression of FlhD_2_C_2_ *in trans* from the p*flhDC* plasmid (Fig. 10A, black bars). Additionally, we determined that the wild-type strain and the otherwise isogenic *phlA* mutant strain showed equivalent invasion capacity (data not shown). These results demonstrate that the expression of the flagellum, but not of the co-regulated PhlA exoenzyme, is a key determinant in the early steps of *Serratia* interaction with epithelial cells. However, the three strains analyzed (wild-type, *flhD* and *phlA*) were equally capable of inducing the EGFP-LC3 autophagic pattern (i.e. punctate distribution) when colocalization of LC3 with the SeCV was monitored. Once again, the autophagy phenotype was observed in both invaded and non-invaded cells 120 min p.i (Fig. 10B, a–d). Interestingly, LC3 puncta was replaced by a cytoplasmic homogeneous fluorescence only in non-invaded cells, provided that extracellular bacteria were eliminated by the action of gentamicin (Fig. 10B, e–h). This observation also correlates with our previous result showing that bacterial *de novo* protein synthesis was required to induce the autophagy phenotype in CHO cells (Fig. 9B and C). These results indicate that the autophagy process is neither induced by flagellar-dependent adhesion of *Serratia* to the host cell nor by the expression of PhlA. They also reinforce the notion that autophagy could be activated by the bacteria from outside the target cell, being the autophagic phenotype sustained at later stages post-infection only in invaded cells.

## Discussion

To understand the mechanisms that *Serratia* uses to invade and establish in the host, we set up an *in vitro* infection assay into epithelial CHO cells and examined events that determine the fate of the pathogen inside a non-phagocytic cell type.

In the present report we demonstrate that *Serratia* is able not only to be internalized but also to replicate within different epithelial cell lines. Once inside CHO cells, bacteria augmented 6-fold its population in the 2 to 4 h p.i. lapse. Afterwards, intracellular counts remained essentially stable up to 8 h p.i. The steady state in bacterial CFUs reached by *Serratia* beyond 4 h p.i. could be interpreted as equilibrium reached between bacterial propagation and cellular elimination mechanisms or to a modulation of bacterial division controlled by bacteria-cell interaction cues. Correlating with these results, small bacteria-containing vacuoles (1–4 bacteria/vacuole) were observed at early times after infection, while large compartments packed with bacteria (>20 bacteria/vacuole) were predominantly visualized at later infection times. These results indicate that *Serratia* manages to counteract or delay the eradication processes that are expected to be physiologically triggered inside the host cell to get rid of the intruder.

Upon cell invasion, autophagy is a key component of the innate host defence that intracellular bacteria must deal with. However, numerous pathogens such as *Coxiella burnetii* [23], [32], *Staphylococcus aureus* [33], or Group A *Streptococcus* (GAS) [10] display sophisticated strategies to evade, hamper or even manipulate the normal progression of the autophagic process to establish a persistent infection.

Remarkably, there is accumulating evidence that, within an invasion event, populations of internalized bacteria can manage to differentially interact with the vesicular traffic pathways, resulting in diverse destinies. This is nicely exemplified by the work of Birgmingham *et al.* [12] where they provide evidences that at least four distinct *Salmonella enterica* intracellular populations coexist: bacteria that reside and proliferate in a modified endosomal compartment avoiding maturation; a population in which the *Salmonella*-containing vacuole (SCV) is damaged by action of bacterial effectors either promoting progression to degrading endolysosomes or being targeted by the autophagy system, and a last population that is able to escape from the damaged SCV to the cytosol.

To understand the events that allow *Serratia* to establish an intracellular replicative niche, we explored the interaction of this bacterium with the autophagic pathway. The induction of the autophagic process, evidenced by EGFP-LC3 recruitment from the cytoplasm to the membrane of the autophagosomes, was detectable as early as 30 min p.i. EGFP-LC3 colocalization with *Serratia* raised from 10% to 55% in the 45 min-6 h post-infection period. LC3 I to lipidated-LC3II conversion triggered by the invasion process was also corroborated by immunodetection assays. By confocal microscopy an autophagic phenotype was also observed in other epithelial cell lines challenged with *Serratia*, showing that it is not a response only restricted to CHO cells.

The intracellular bacteria targeted by autophagy are expected to be removed by degradation when the autophagic vesicle containing the unwanted cargo is delivered to the lysosome, forming the autophagolysosome. This process involves a stepwise progression in which the initial phagophore fuses with endocytic vesicles. Rab GTPases are essential in regulating the generation, transport, recognition and fusion of vesicles. In particular, the GTPase Rab7, that catalyzes the homotypic fusion of late endocytic vacuoles, has been demonstrated to be required not only for the maturation of canonical autophagosomes [16], [17] but also in the formation of non-canonical autophagosomes that surround intracellular pathogens such as GAS [18]. In the *Serratia*-containing vacuoles (SeCV), Rab7 followed an acquisition kinetic similar to LC3 recruitment. Rab7 acquisition was detected early after internalization and decorated the SeCV at all times analyzed along the invasion assay. This correlation strongly suggests that this GTPase participates in the biogenesis of the autophagosome-like vacuole that sustains *Serratia* intracellular life. We can conjecture that the large bacteria-packed autophagosomes observed mostly at prolonged times post-infection could be the result not only of active intravacuole bacterial replication but also of autophagosome-autophagosome fusion mediated by Rab7.

However, an arrest of the fusion events of the SeCV with late endo/lysosomal compartments was evidenced because in an estimated 85 to 90% of the total SeCV labelled by the LC3 autophagic marker, neither colocalization of the bacteria with the acidotropic Lysotracker marker nor with hydrolyzed DQ-BSA was detected even at six hours post-infection. Therefore, the expected progression to a functional autophagolysosome was somehow blocked or delayed by an intracellular *Serratia* population. As a consequence, we can conclude that *Serratia* is able to occupy a niche where the adverse action of acidic pH and lytic enzymes is circumvented.

In contrast to the predicted outcome, when autophagy was induced by starving the CHO cells, no enhancement of intracellular survival of *Serratia* was observed. Moreover, blockage of *Serratia*-mediated autophagy activation by action of the PI3K inhibitor wortmannin was also ineffective, although this inhibitor drastically diminished the intracellular levels of CFUs recovery when added previous to the bacterial invasion with the aim of blocking the process extracellularly triggered by *Serratia*. Wortmannin was, as expected, proficient in blocking the starvation-activated autophagic process, but it was ineffective once *Serratia* induction has been triggered. The overall effect of wortmannin is to block autophagy because class III PI3Ks act downstream of class I PI3Ks in the autophagic process. However, wortmannin has been found to be an inhibitor of class III (autophagy stimulator) as well as class I PI3K (autophagy negative regulator) [9], [34], while also inhibiting other members of the PIK family, such as mTOR and the DNA-dependent protein kinase as well [35]. Taking also into account that PI3Ks are involved in other membrane trafficking processes, including formation of phagocytic cups and micropinosomes [36], our results allow us to infer that *Serratia* is internalized in the CHO cells by a process in which PI3K activity is required and that the autophagic process induced by *Serratia* is able to bypass the involvement of PI3K. On the other hand, the invasion assays performed with *Atg5* knock-down cells showed that Atg5, component of the Atg12-Atg5-Atg16 complex which acts as a ligase for LC3-phosphatidylethanolamine conjugation, is essential for the autophagy activated by *Serratia*. Mestre *et al* [33] recently demonstrated that, in the *Staphylococcus aureus* infection, α-toxin-dependent activation of autophagy is independent of PI3K activity. In addition, a recent work from Kageyama *et al* [37] shows that LC3 and the Atg16L complex are recruited to the autophagosome-like membrane structure that surrounds *Salmonella* inside the *Salmonella* containing-vacuole, in a PI3K-independent way. Together, these observations contribute to indicate that, depending on the specific pathogen, autophagy triggered by bacteria might implicate non-canonical pathways, in a process still barely understood.

Additionally, we show that the integrity of the flagellar biogenesis cascade is required for the early interaction of *Serratia* with CHO cells. As expected, the adhesion impairment of the non-flagellated strain essentially abrogated the subsequent entry of the bacteria into the cells. Nonetheless, neither the lack of flagellum biogenesis nor the inhibited expression of the PhlA phospholipase (the only *Serratia* exoprotein that is known to depend on the flagellar regulatory cascade for its expression and secretion) prevented the induction of autophagy. This result clearly shows that even when the bacterium-cell interaction stage that precedes invasion is inhibited, autophagy can still be activated by extracellular *Serratia*. Furthermore, when bacterial protein synthesis was halted prior and along the invasion assay, the induction of the autophagic phenotype was prevented. Besides, the autophagic phenotype of non-invaded CHO cells was lost as soon as metabolically active extracellular bacteria were killed by gentamicin action. This result indicates that the autophagocytic response is induced by metabolically active bacteria, previous to internalization. Reinforcing this observation, heat-killed bacteria were capable neither to induce autophagy nor to invade the cells, ruling out the involvement of bacterial heat-resistant structures such as the lipopolysaccharide in autophagy activation.

In addition, we show that an early *Serratia* invasion event is severely stalled by preventing vacuole acidification, suggesting that a bacterial determinant that requires a low pH for intracellular action might be implicated. In this context, it has been demonstrated that diverse bacterial toxins that enter their target cell by receptor-mediated endocytosis, require acidification for their processing and activation, being its effect prevented by treatment with bafilomycin A1 or NH_4_Cl [38], [39]. Alternatively or in addition, the increase of the vacuoles luminal pH can secondarily cause changes in eukaryotic receptors turn-over, in the conformation of proteins or in the affinity between receptors and ligands required for invasion (reviewed in [40]). We are currently analyzing the identity and mechanism of action of the virulence factors required for entry and for the promotion of the autophagic process.

In light of our results, it is tempting to speculate that the extracellular action of a virulence factor previous to the internalization process activates a non-canonical autophagic pathway as a strategy that leads to the subsequent generation of a specialized vacuole that supports the intracellular life of *Serratia*. Once inside the SeCV, the bacterium would delay or preclude the interaction of the autophagosome-like vacuole with the lysosomal compartment. As a consequence, *Serratia* alters the normal outcome of the autophagosome, building a propitious environment for survival and proliferation. These findings also put forth that *Serratia marcescens* invasion constitutes an ideal system to further explore how an opportunistic pathogen is proficient to manipulate autophagy throughout the infective process.
